# Why improving the measurement and monitoring of essential maternal and newborn health interventions matters

**DOI:** 10.7189/jogh.10.020106

**Published:** 2020-12

**Authors:** Jennifer Harris Requejo, Shams el Arifeen

**Affiliations:** 1 United Nations Children’s Fund, Data and Analytics Section, Division of Data, Analytics, Policy and Monitoring, UNICEF USA, New York, USA; 2 Maternal and Child Health Division, International Centre for Diarrhoeal Disease Research, Dhaka, Bangladesh

The goal of ending preventable maternal and newborn mortality is achievable. Many maternal and newborn deaths can be averted through family planning services, and the timely provision of high quality care throughout pregnancy, childbirth, and the postnatal period. Yet, progress in increasing universal access to these life-saving interventions has been uneven and slower than we hoped. There are still approximately 2.5 million newborn deaths and 300 000 maternal deaths per year, most occurring in countries with the highest resource constraints and weakest health care infrastructures [1,2].

The positive news is that these stark numbers have not gone unnoticed, and great strides have been made in identifying essential packages of care for saving the lives of mothers and their newborn babies. Global consensus has also been reached on a core set of indicators for assessing progress in scaling-up these essential packages. Targets for reducing maternal and newborn mortality are in the Sustainable Development Goal (SDG) Framework, and many of these core indicators are embedded within other global accountability frameworks such as the Global Strategy for Women’s, Children’s and Adolescents’ Health (2016-2030), Countdown to 2030, Every Newborn Action Plan, and Ending Preventable Maternal Mortality.

But, why does it matter if maternal and newborn health related indicators feature prominently in global accountability frameworks? The simple answer is the maxim we know so well: What gets measured gets done. And this translates into all of us doing our part to make it possible for every woman to have a healthy pregnancy and positive birth outcome for herself and her newborn baby.

The inclusion of maternal and newborn health indicators in global accountability frameworks has spurred a surge of data collection efforts in recent years – new questions and modules have been added, for example, to large-scale population-based surveys such as the UNICEF supported Multiple Indicator Cluster Surveys (MICS) and the USAID supported Demographic and Health Surveys (DHS), and to health facility assessments such as MEASURE’s Service Provision Assessment, and WHO’s Service Availability and Readiness Assessment. There are also ongoing efforts to provide guidance to countries, through a set of modules (DHIS2 modules) produced under the umbrella of the Health Data Collaborative, on a standard set of indicators to collect through their routine health information systems.

All these data collection efforts have greatly increased the availability of data on essential maternal and newborn health interventions. The aim of this supplement is to take stock of where we are now: Are these efforts enough to meet global and national monitoring needs for maternal and newborn health? What else needs to be done to improve the measurement of maternal and newborn health indicators so that we all can be held to account for achieving the ambitious SDG targets? This supplement is organized into three sections. The first provides an overview of the data landscape for essential maternal and newborn health interventions. The second section includes papers that focus on the policy and programmatic implications of accurately measuring and regularly monitoring key maternal and newborn health indicators. These studies demonstrate what can be learned from analyses of available data about progress in individual and in groups of countries. They highlight where improvements are being made, where more work is needed, and context specific opportunities for increasing both the supply of and demand for essential maternal and newborn care. The set of papers in the third section highlights methodological work completed or under way on improving the measurement of maternal and newborn interventions and how this work helps address existing data gaps.

Several actionable messages emerge from the collection. These include a recommendation to increase data collection approaches that capture information about effective coverage and the quality of care. The study by Carvajal-Aguire et al., for example, revealed large data gaps in 20 countries in sub-Saharan Africa on the content of health services provided [3]. One such approach proposed to address these gaps is to improve the interoperability of household and health facility surveys, and to develop robust methods for linking different survey modalities [4]. Improving the quality of data captured through country health information systems would also enable more real time assessments of service provision, equipping program managers with the information they need to better determine where resources should be allocated and where course correction is needed.

Although the escalation in data availability on maternal and newborn health interventions is a laudable achievement, studies in this collection show that further work is needed on harmonizing data collection efforts to improve comparability across them. The study by Amouzou et al., for example, found that the MICS and DHS use different questions and methodologies for capturing information on postnatal care which reduces comparability across the two survey programs and the ability to develop time trends [5]. Other authors point out that data are still lacking on newborn practices which must be remedied, and they present the limitations of using early initiation of breastfeeding as a proxy measure for essential newborn care [6]. The individual country papers show how data helps reveal context specific issues and the need to tailor intervention delivery strategies accordingly.

This supplement is timely given the ambitious global development agenda and the growing awareness of maternal and newborn survival as essential to its achievement. It shows the power of data to: 1) inform us about how well we are doing in improving coverage of essential maternal and newborn interventions within and across countries, and 2) why investments in the measurement agenda are critical for increasing our ability to know what we need to know about the success of policies and programs in saving maternal and newborn lives.

## References

[1] UN-interagency Group for Child Mortality Estimation. 2018. Levels & Trends in Child Mortality: Report 2018. New York: United Nations Children’s Fund; 2018.

[2] WHO, UNICEF, UNFPA, World Bank Group and the United Nations Population Division. Trends in Maternal Mortality: 1990 to 2015. Geneva: World Health Organization; 2015.

[3] Carvajal-Aguirre L Amouzou A Mehra V Ziqi M Zaka N Newby H Gap between contact and content in maternal and newborn care: An analysis of data from 20 countries in sub–Saharan Africa. J Glob Health. 2017;7:020501. 10.7189/jogh.07.020501 29423178 PMC5804037

[4] Carvajal-Aguirre L Mehra V Amouzou A Khan SM Vaz L Guenther M Does health facility service environment matter for the receipt of essential newborn care? Linking health facility and household survey data in Malawi. J Glob Health. 2017;7:020508. 10.7189/jogh.07.020508 29423185 PMC5804506

[5] Amouzou A Mehra V Carvajal-Aguirre L Khan SM Sitrin D Vaz LME Measuring postnatal care contacts for mothers and newborns: An analysis of data from the MICS and DHS surveys. J Glob Health. 2017;7:020502. 10.7189/jogh.07.020502 29423179 PMC5804502

[6] Sitrin D Perin J Vaz LME Carvajal-Aguirre L Khan SM Fishel J Evidence from household surveys for measuring coverage of newborn care practices. J Glob Health. 2017;7:020503. 10.7189/jogh.07.020503 29423180 PMC5804503

